# Sharing of science is most likely among male scientists

**DOI:** 10.1038/s41598-017-13491-0

**Published:** 2017-10-10

**Authors:** Jorg J. M. Massen, Lisa Bauer, Benjamin Spurny, Thomas Bugnyar, Mariska E. Kret

**Affiliations:** 1 Department of Cognitive Biology, University of Vienna, Vienna, Austria; 2 Cognitive Psychology Unit, Institute of Psychology, Leiden University, Leiden, The Netherlands; 3 Leiden Institute for Brain and Cognition (LIBC), Leiden, The Netherlands

## Abstract

Humans are considered to be highly prosocial, especially in comparison to other species. However, most tests of prosociality are conducted in highly artificial settings among anonymous participants. To gain a better understanding of how human hyper-cooperation may have evolved, we tested humans’ willingness to share in one of the most competitive fields of our current society: academia. Researchers were generally prosocial with 80% sharing a PDF of one of their latest papers, and almost 60% willing to send us their data. Intriguingly, prosociality was most prominent from male to male, and less likely among all other sex-combinations. This pattern suggests the presence of male-exclusive networks in science, and may be based on an evolutionary history promoting strong male bonds.

## Introduction

Prosociality has been proposed as a hallmark of humanity^1^, and studies on prosociality in our closest living relatives have long corroborated the idea that such behavior is uniquely human (e.g.^2,3^ but see^4^). A major drawback is that experimental methods for studying prosociality differ dramatically, particularly between human and non-human studies, and that many experiments lack ecological relevance^5^. For example, in humans most of these tests are computerized cooperation tasks or public goods games performed in peer groups of college students who anonymously make decisions about an interaction partner they do not know and will never need to interact with in the future. However, that is not the social environment on which selection acted during the evolution of humans’ so-called “hyper-cooperation”^6^, and recent studies showed that when for example some sort of competition^7^ or hierarchy^8^ is added to the original cooperation or public goods games, human prosociality/cooperation breaks down easily. Moreover, these economic games are relatively artificial and their ecological relevance is rather unclear^9^. Therefore, here we tested human prosociality in our own everyday situation, which may reflect the competitive environment in which prosociality possibly evolved. As most of us are not hunting animals on the savanna anymore, we choose a contemporary competitive environment, namely academia.

Science is becoming one of the most competitive professional fields^10^. Whilst the percentage of young people that finish a Ph.D. has doubled over the last two decades, the amount of available jobs in science has not. Tenured positions are becoming increasingly scarce, and additionally, around the globe governmental funding for research projects has either plateaued or declined^11^. As a consequence, it has been suggested that researchers are not always willing to share their data, since they are scared of being ‘scooped’ by their competitors^12^; i.e. another scientist publishes first, and therefore, gets the credit for a theory/hypothesis or results instead of the one sharing it initially. Here, we investigated researchers’ willingness to share their research in detail. In particular, we tested the effect of the scientists’ career status, the costs and benefits of sharing, and the effect of the different sex-combinations of requester and responder.

We were interested in the effect of different sex combinations because men and women are known to differ in the specific social contexts that trigger prosocial acts. There are some reports that women are kinder than men (e.g.^13^), yet in general, men and women do not differ in their overall amount of cooperation or prosociality (meta-analyses on social dilemmas^14^; and on economic games^15^). However, differences start to emerge when a social or emotional context is provided or when they can take their time and freely think over a social decision^15^. Academia is far from a neutral environment and competition in science is much harsher for women than for men. For example, women are expected to be less brilliant across academic disciplines^16^, are less likely to be hired for faculty positions^17–19^ or to receive funding^18^, and hence, are significantly underrepresented at the professor level^20^.

Moreover, sex-combinations may influence cooperation in different ways. For example, male-male combinations are reported to be more cooperative than female-female combinations^14,21^. Such male-male alliances may have a cultural background in so called “Old Boy” networks^22^, and a more evolutionary history in which particularly male bonds were promoted to cope with inter-group conflicts; i.e. the Male Warrior Hypothesis^23^. Alternatively, males may be more prosocial towards females to impress them^24^, which may even lead to competitive altruism among those men^25^ and may increase mating success^26^. Similarly, high-ranking individuals (cf. leaders) may signal their status by providing benefits to their group members^27^. Alternatively, low status may enhance prosocial behavior^28^, and for example, in highly despotic primate species low-ranking individuals may use prosociality towards higher-ranking individuals to gain future tolerance and or support^29^. Furthermore, the status of scientists does influence how much competition they experience, since older/higher-ranking scientists may already have tenure, and their chances to receive grants are much higher^11^. Moreover, sex and status may interact since around the world males are culturally associated with power^30^. Finally, costs of acting prosocial may influence decisions to do so (e.g.^31^).

Specifically, in this study in a low-cost condition we sent an email to researchers who had recently published in the fields of comparative psychology and social cognition, requesting a PDF of one of their recent publications. We chose these two fields to ensure plausibility of the requests with regard to the field in which we (the authors) work. Emails were sent by two master students (LB: ♀ & BS: ♂) and two post doctoral researchers (MEK: ♀ & JJMM: ♂, similar in age, year of PhD, H-index and international network), all using a university email-address, and the different participants were assigned semi-randomly to those four researchers (each an equal share); i.e. randomly, yet trying to keep the sex ratio of participants equal among all four researchers and while making sure that the two post-docs did not know the participants they emailed personally (to avoid favors among friends: cf.^32^). Second, in the high-cost condition, we semi-randomly choose a subset of those former low-cost participants; i.e. randomly and independent from their previous response, yet making sure that they had published a data-paper of which the data would be suitable for a potential meta-analyses. These participants were then emailed by one of the other researchers (again semi-randomly assigned: as above) and were asked whether they in principle were willing to share data of one of their studies for a meta-analyses on a related topic. Note that we deliberately did not ask for the data yet, and that we also did not receive any data. Also note that we did not offer any co-authorships or anything else. Informed consent was acquired afterwards; i.e., all participants received an email explaining the purpose of the study and were allowed to retract their responses from the sample. For the full text of all three emails please see the supplementary information (SI). In total 288 out of 292 participants (142 females with average h-index of 9.2 (SD = 9.3) and 146 males with an average h-index of 16.2 (SD = 15.5); from 32 countries all over the world) allowed us to use their responses with regard to our request for a paper for analyses, and 106 out of 110 participants (49 females with average h-index of 22.6 (SD = 15.2) and 57 males with average h-index of 13.7 (SD = 13.4) allowed us to use their responses with regard to the data request for further analyses.

## Results

Using a Generalized Linear Mixed Model with a binomial distribution and a logit link function we analyzed what influenced the willingness of participants to either share one of their papers or to share data; i.e., yes or no, where no response was lumped with negative responses, since the latter did not happen in response to a paper request and only 10 times in response to a data request. As fixed factors we entered the sex of the participant, the sex of the requester, the interaction between the participant’s sex and that of the requester, whether the requester was a student or a postdoctoral research fellow, and the condition (low cost = paper request; high cost = data request). As covariates we entered the h-index of the participant (range: 0–97), and the difference in h-factor between requester and participant. Additionally, we entered to the model the 2-way interactions between the participants’ sex and both their h-index and the h-difference, and all (2-, or 3-way) interactions of all the above with condition. Since the participants in the high-cost condition were a subset of those researchers that already participated in the low-cost conditions, we controlled for repeated measures by structuring our data as being nested in participant ID (number) and added participant number as a random factor to the model. We ran the full model described above (see SI for results) and reduced models and chose the best fitting model based on comparisons of the corrected Akaiki Information Criteria of those models. The best fitting model contained only three factors; i.e. ‘Sex of Participant’, the 2-way interaction between ‘Sex of Participant’ and ‘h-index of Participant’, and the 3-way interaction of ‘Sex of Participant’, ‘Sex of Requester’, and ‘Condition’, and revealed that all three had a significant effect on the willingness to share.

**Figure 1:**
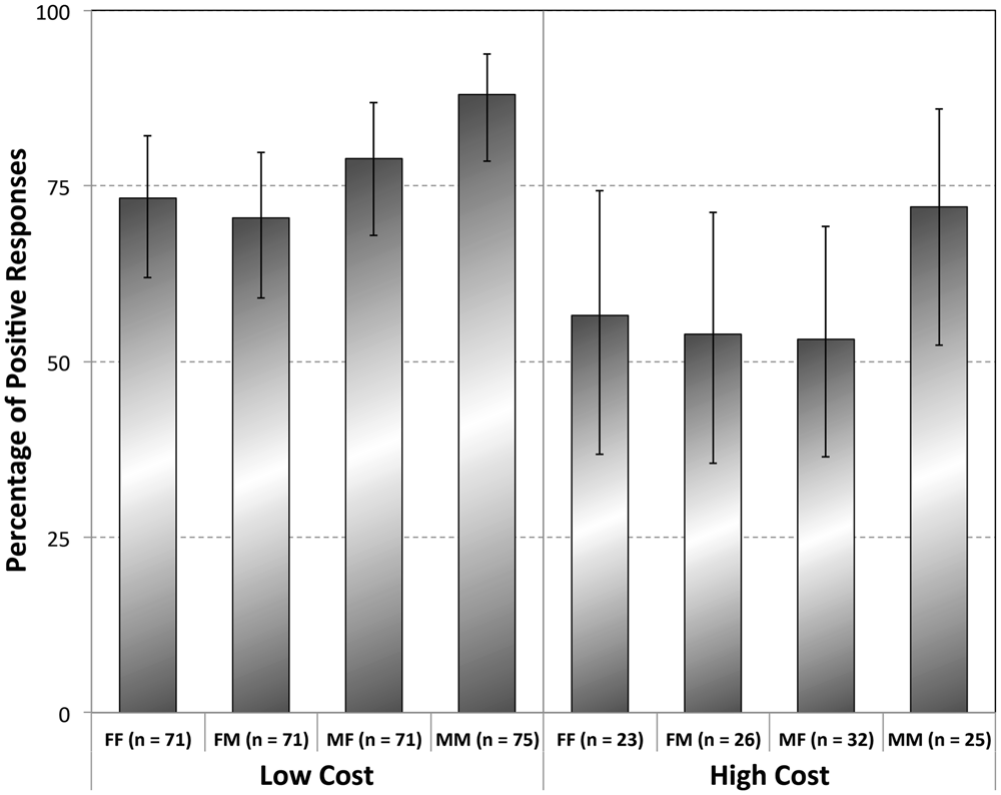
Percentage ±95% CI of positive responses of female participants to requests of females (FF), of females to males (FM), of males to females (MF), and of males to males (MM), when the request was either a paper (Low Cost) or data (High Cost).

First, we find that males are more likely to share their science than females (β = 0.88, SE = 0.5, F_1, 384_ = 8.16, P = 0.005; Fig. 1). Second, the interaction effect between ‘Sex of Participant’, ‘Sex of Requester’, and ‘Condition’ (F_6, 384_ = 3.27, P = 0.004; Fig. 1) shows that the difference between males and females becomes particularly evident when those males got a request from a male requester. Post-hoc comparison of the number of positive and negative responses revealed that, after Bonferroni corrections, specifically males were significantly more likely to share with other males in comparison to males with females (Chi^2^ = 13.69, P_corrected_ = 0.001), females with males (Chi^2^ = 22.82, P_corrected_ = 0.00001), and females among each other (Chi^2^ = 14.86, P_corrected_ = 0.0007), whereas there were no differences between the other sex combinations (Fig. 1). Moreover, this difference is most apparent in the high-cost condition, and a post-hoc comparison of the likelihood of positive responses of males towards female requesters and male requesters, indeed shows that this difference is significantly higher (Chi^2^ = 11.64, P = 0.0009) in the high-cost condition compared to the low-cost condition (Fig. 1). Additionally, we validated our use of two different conditions, as indeed participants were more likely to share a paper (low-cost: 77.8% of participants responded (positively)) than data (high-cost: 58.5% of participant responded positively)(Comparison of the number of positive and negative responses between high-cost and low-cost: Χ^2^ = 14.49, P = 0.0001).

**Figure 2:**
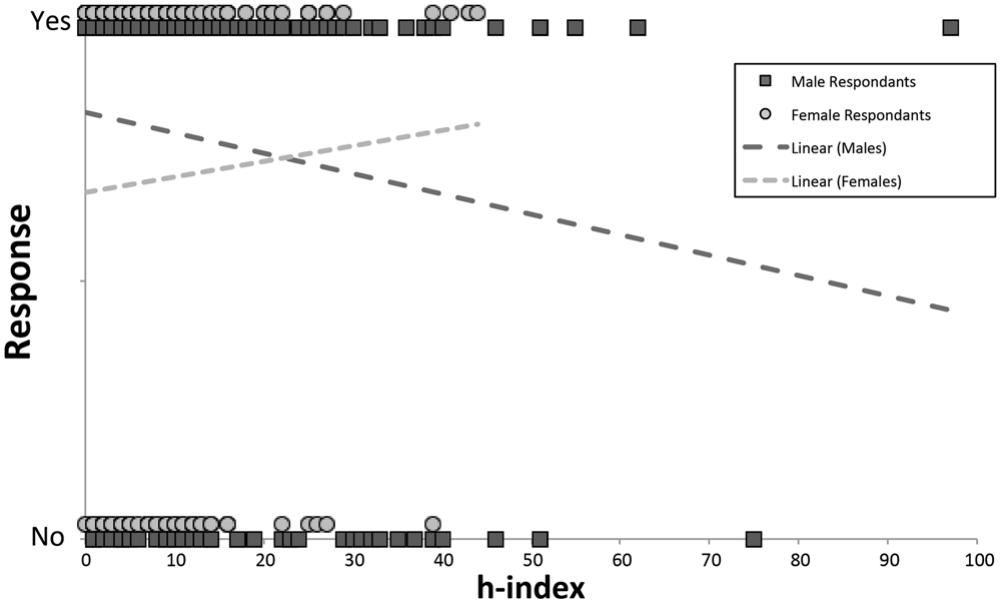
Positive (yes) and negative/no (no) responses of males (dark) and females (light) with increasing h-index. Trend-lines represent likelihoods of positive responses in relation to h-index for males and females separately.

Third, the interaction effect between the sex of the participants and their h-index (F_2, 384_ = 3.27, P = 0.049), shows that for males the likelihood of responding positively to a request for a paper or data decreases with increasing h-index (β = −0.029, SE = 0.012, P = 0.017; Fig. 2), whereas for females such an effect was not observed (β = 0.012, SE = 0.02, P = 0.564; Fig. 2).

Finally, we tested whether for those participants that participated in both the low- and the high-cost condition, their responses in the low-cost condition had any effect on their response in the high-cost condition, using a GLMM with a binomial distribution and a logit link function in which we included participants’ ID as a random effect. We find that there is a non-significant trend that those participants that responded positively in the low-cost condition were more likely to also respond positively in the high cost condition (β = 0.83, SE = 0.49, F_1, 101_ = 2.9, P = 0.092), or vise versa that those participants that did not respond in the low-cost condition were more likely to not respond or respond negatively in the high-cost condition.

## Discussion

We show that, even though science is becoming increasingly competitive^9,10^, in general the scientists we addressed were very willing to share their work. Almost 80% of them send us a reprint of a recent paper, and almost 60% were willing to share their data, even though they were not offered anything in return. However, the likelihood of sharing depended heavily on the sex of the participant, and on the combination of both the requester’s and the responder’s sex; i.e., particularly males that received a request from another male were most likely to share (a difference of ~15% with all other sex-combinations). Moreover, for males the likelihood of sharing also depended on their h-index.

The sharing of one’s own papers poses very little cost (time), increases exposure and might even lead to a citation. However, the cost-benefit ratio of sharing data is very different as it most likely took very much time to collect that data and a single citation would be the only payoff in this scenario, and there may even be additional costs of a scoop^12^. And although indeed responses were lower when costs were higher, corroborating earlier findings in non-human primates^31^, nevertheless, on average 58.5% of the participants happily shared their data. This is in line with the general view on human prosociality^1^, but also with a recent trend where many journals ask authors to upload their data in data repositories even before the acceptance of their paper (e.g. the PLoS and Royal Society publishing groups) which of course demands high levels of trust that other scientists will not use that data for their own sake only.

Whereas overall females responded less than males, males only responded more positively when the request came from another male and responded in a similar rate as females when the request came from a female. Consequently, our results do not corroborate any sexually selected motives for sharing of males^24–26^. A possible explanation for the male pattern may be that among male academics there is a network at play, in which they favor each other much like “Old Boy” networks^22^. The lower response rate of females may be due to the increased competition that females experience in academia compared to males^16–20^, and the possible increased chance of being “scooped”^12^. Alternatively, as our response variable also included a ‘no response’, it may well be that females are not less willing to share, but simply have less time to answer e-mails due to their increased competitive environment. However, a lack of time does not explain the differentiation in response rates between low cost and high cost, nor the pattern that males share most with other males. A time-constraint may explain the effect that with increasing h-index, males become less likely to respond, because time-constraints tend to increase with status/the advancement of one’s career. Alternatively, higher rank may create distance to others^30^ and therefore a lower likelihood of responding to others’ requests. We did not find a similar effect for females, yet this may be due to the fact that in our *random* sample, females on average had a much lower h-index (9.2 vs. 16.2) and that the range of men’s h-indexes (0–97) was more than twice as large as that of females (0–43), facts that themselves corroborate the reported male biases in science^16–20^.

The observed male-male pattern may have its roots in evolutionary history. Due to the increased occurrence of intergroup conflict, males became more strongly bonded to secure and maintain coalitionary support in such conflicts^23^. Whereas the “Male Warrior Hypothesis” originally described strong male bonds in relatively small in-groups providing coalitionary support against out-groups, a recent study showed that derived features of it persist even in larger contemporary communities; i.e., male professional sports players affiliate longer with their opponents after a within-group conflict (a sports match) than females, regardless of culture or type of sports^33^. Similarly, in our study the participants may have perceived to be part of the same scientific community and males’ bonds or their willingness to bond may have led them to share more with each other than with females, and also more than females share themselves.

In sum, we show that also in a competitive environment scientists working in comparative psychology can be prosocial, and that this is specifically true for male-male donor and recipient combinations. Such male-male oriented prosociality amongst scientists suggests male-exclusive networks in academia, which may originate from an evolutionary history that promoted strong male-male bonds. More research will be needed to truly pin-point the origin of these differences and to investigate whether these results can be generalized to other fields and disciplines, or even humans in general.

### Ethics

This study followed the guidelines on human experimentation of the Declaration of Helsinki and was approved by the Ethical Committee of the University of Vienna (reference number: 00131).

## Electronic supplementary material

Supplementary Information
