# Supplementary material for: Sharing of science is most likely among male scientists
Source: Sci Rep. 2017 Oct 10;7:12927. doi: 10.1038/s41598-017-13491-0 (PMC5635125; doi:10.1038/s41598-017-13491-0)

**Supporting Information for**

**Sharing of science is most likely among male scientists.”**

*By* Jorg J. M. Massen, Lisa Bauer, Benjamin Spurny, Thomas Bugnyar & Mariska E. Kret

**Content:**

Text e-mail requesting paper 2

Text e-mail requesting data 3

Text e-mail post-hoc consent 4

Full model on likelihood of sharing 5

subject: request for reprint

Dear Mr. /Mrs…..

I would be grateful for a reprint of your paper (“title of the paper”).

Many thanks in advance,

yours sincerely,

(“First and Surname of requester”)

subject: request for data for a meta-analysis

Dear Mr. /Mrs……..,

We are currently looking into the possibility of a meta-analysis on (“topic of meta-analysis”). Therefore, we are investigating whether we can obtain enough data to make this a relevant study.

Your study on (“Title of the study”) fits our requirements, and therefore we would like to ask you at the moment whether in principle you would be willing to share data from that study. If, in total we would be able to get enough data to make this project feasible we would then contact you again, with regard to the details of the study and the actual data required.

Please let us know whether you would be willing to share your data or not.

Many thanks in advance,

yours sincerely,

(“First and Surname of requester”)

subject: Consent for the use of your response to a request as data in an empirical study?!

Dear Mr. /Mrs……..,

(“Thank you very much for your response to our data-request.”)

We have to inform you (, however,) that you participated in a study on prosociality;

(“i.e., recently you received the request to share one of your papers by a member of our research team (see below).”)

(“i.e., recently you received the request to share data, and prior to that you were also asked to share one of your papers by a different member of our research team.”)

Although, we are indeed interested in your work, the sole purpose of our requests was to see whether you would or would not reply to it, and if you did, whether your reply was positive or negative.

Note that this study received oversight from and was approved by the Ethical Committee of the University of Vienna (projectnr. 00131).

What is the aim of this study?

In this study, we investigate pro-social behaviour among researchers. Recently, there has been a surge in research on prosocial behaviour in animals, mainly to investigate the evolution of what is sometimes called human ‘hyper-cooperation’. Here, we question how cooperative humans actually are. Although there is a vast body of literature on human prosociality, most of these studies have been conducted on children and/or in a relatively uncompetitive context. Therefore, we aimed to study how prosocial adult humans are in a competitive context, namely in contemporary science. In addition, we study how relative rank differences among researchers might influence prosociality. In particular, the willingness you showed to share your paper was used as indicator for pro-social behaviour.

To further analyze our data, we use publically available personal data (your gender and h-index) that we obtained from the internet. Nevertheless, we will make sure that after initial coding all data will be anonymous and that all personal data will be exclusively used in this study and safely stored at the university.

We write you to allow you the possibility to withdraw your (response) data from our study. Therefore, if you are unwilling to participate and want us to exclude you from the study please answer this mail or directly contact the head of the study: Dr. Jorg Massen, University of Vienna (jorg.massen@univie.ac.at).

We highly appreciate your effort.

Best regards,

(“Names of all requesters”)

**Full model on likelihood of sharing**

**Table S1** Test of fixed effects: i.e. sex of the participant (SexPart), the sex of the requester (SexReq), the interaction between the participant’s sex and that of the requester, whether the requester was a student or a postdoctoral research fellow (StudPostDoc), and the condition (Cost: low cost = paper request; high cost = data request), and the covariates h-index of the participant (HindexPart), and the difference in h-factor between requester and participant to the model (Hdifference). Additionally, the 2-way interactions between the participants’ sex and both their h-index and the h-difference, and all (2-, or 3-way) interactions of all the above with condition.


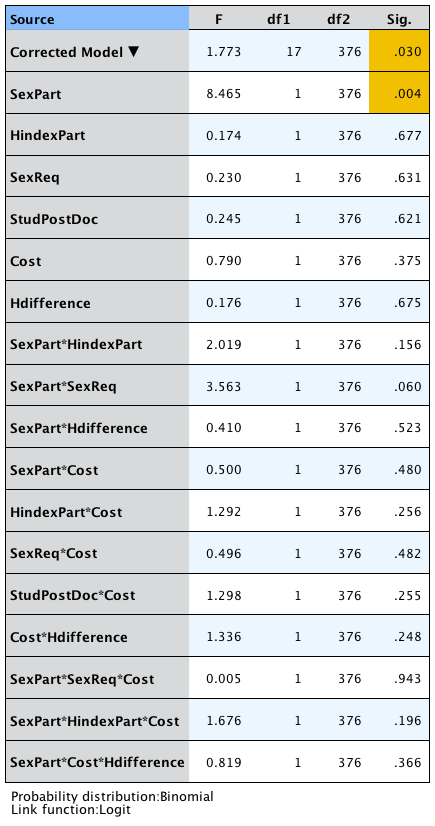


**Table S2** Standardized estimates ± SD of full model


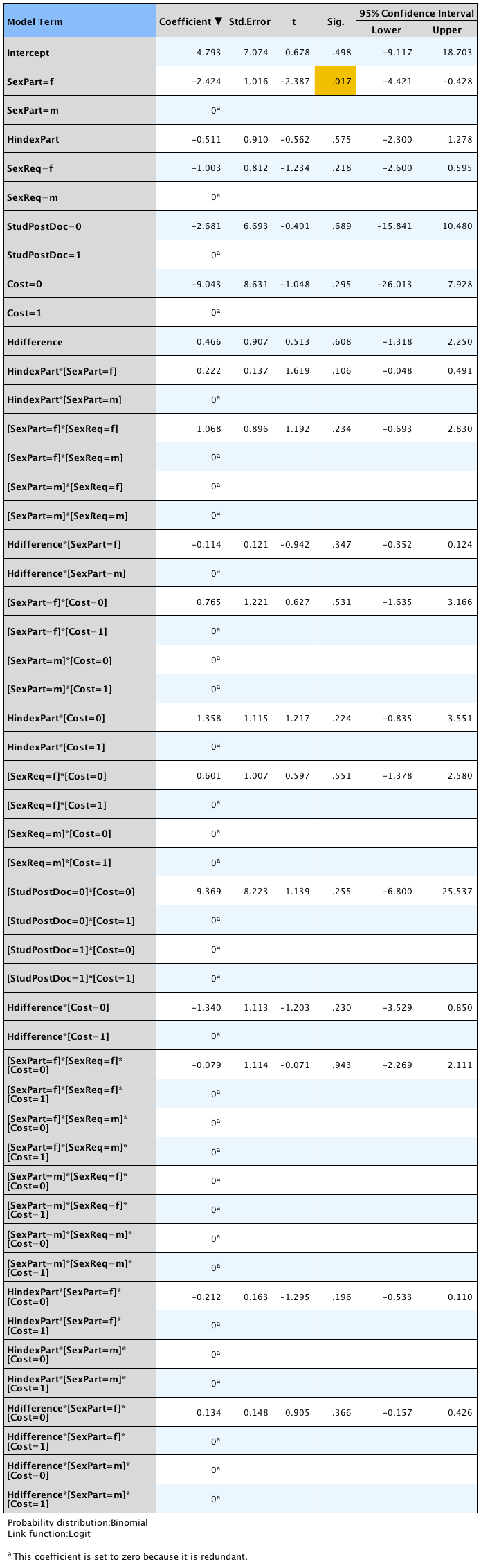


Continues on next page


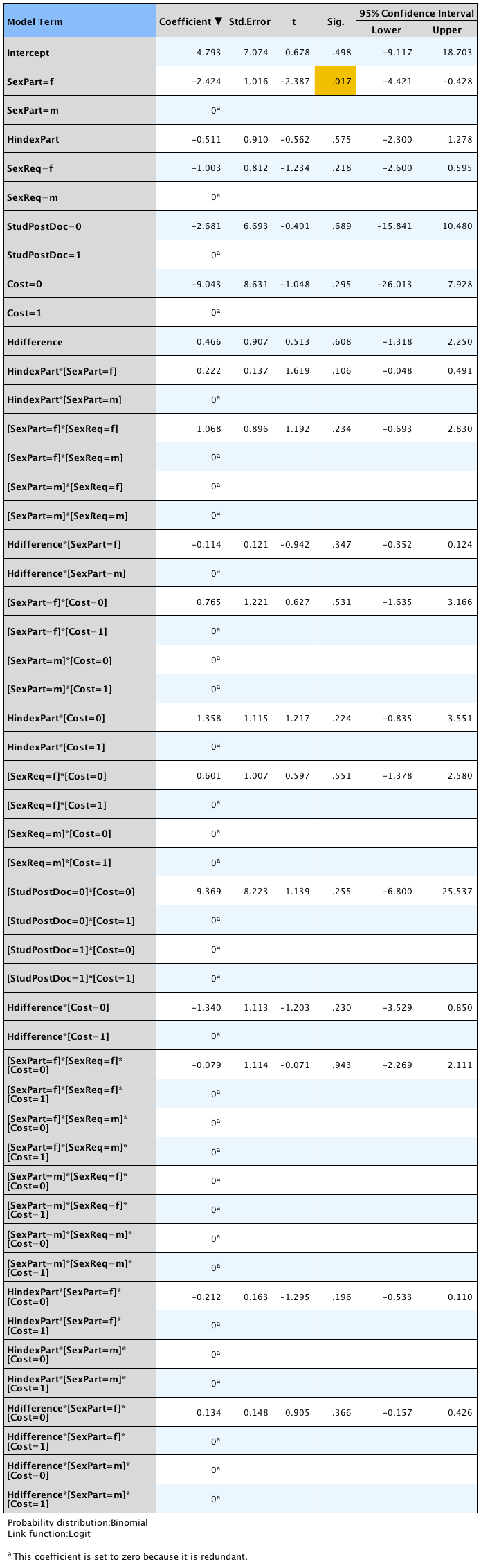

Supplement: Supplementary file 1 — Supplementary Information [file 41598_2017_13491_MOESM1_ESM.doc]
